# Neural Correlates of Semantic Prediction and Resolution in Sentence Processing

**DOI:** 10.1523/JNEUROSCI.2800-16.2017

**Published:** 2017-05-03

**Authors:** Luigi Grisoni, Tally McCormick Miller, Friedemann Pulvermüller

**Affiliations:** ^1^Brain Language Laboratory, Department of Philosophy and Humanities, Freie Universität Berlin, 14195 Berlin, Germany,; ^2^Berlin School of Mind and Brain, Humboldt Universität zu Berlin, 10099 Berlin, Germany, and; ^3^Einstein Center for Neurosciences, 10117 Berlin, Germany

**Keywords:** grounded cognition, semantic processing, semantic readiness potentials

## Abstract

Most brain-imaging studies of language comprehension focus on activity following meaningful stimuli. Testing adult human participants with high-density EEG, we show that, already before the presentation of a critical word, context-induced semantic predictions are reflected by a neurophysiological index, which we therefore call the semantic readiness potential (SRP). The SRP precedes critical words if a previous sentence context constrains the upcoming semantic content (high-constraint contexts), but not in unpredictable (low-constraint) contexts. Specific semantic predictions were indexed by SRP sources within the motor system—in dorsolateral hand motor areas for expected hand-related words (e.g., “write”), but in ventral motor cortex for face-related words (“talk”). Compared with affirmative sentences, negated ones led to medial prefrontal and more widespread motor source activation, the latter being consistent with predictive semantic computation of alternatives to the negated expected concept. Predictive processing of semantic alternatives in negated sentences is further supported by a negative-going event-related potential at ∼400 ms (N400), which showed the typical enhancement to semantically incongruent sentence endings only in high-constraint affirmative contexts, but not to high-constraint negated ones. These brain dynamics reveal the interplay between semantic prediction and resolution (match vs error) processing in sentence understanding.

**SIGNIFICANCE STATEMENT** Most neuroscientists agree on the eminent importance of predictive mechanisms for understanding basic as well as higher brain functions. This contrasts with a sparseness of brain measures that directly reflects specific aspects of prediction, as they are relevant in the processing of language and thought. Here we show that when critical words are strongly expected in their sentence context, a predictive brain response reflects meaning features of these anticipated symbols already before they appear. The granularity of the semantic predictions was so fine grained that the cortical sources in sensorimotor and medial prefrontal cortex even distinguished between predicted face- or hand-related action words (e.g., the words “lick” or “pick”) and between affirmative and negated sentence meanings.

## Introduction

Current theories of brain function emphasize the importance of predictions for perception, action, and language processing (Egner et al., 2010; Friston and Frith, 2015). In language understanding, we frequently know what speakers intend to say before they complete their utterances (Sacks et al., 1974), and even single words can be identified before their end (Marslen-Wilson, 1987). Still, most experimental studies on language focused on processes following, not preceding, the critical expected words (Schwanenflugel and Shoben, 1985; Van Petten and Luka, 2012). The well known N400 (negative-going peak at ∼400 ms) event-related brain potential (ERP; Kutas and Federmeier, 2011) reflects the degree to which critical words are semantically expected in a given context, thus possibly indexing the level of context-induced preactivation of word circuits (DeLong et al., 2005; Otten et al., 2007; Ito et al., 2016). Although the N400 is informative about predictive language comprehension, it follows the (un)expected words and, therefore, the point in time when predictions first arise. If semantic predictions determine the way we process language, a direct neurophysiological index of meaning expectancy preceding the critical linguistic items will be of crucial importance. Recent studies reported neurophysiological correlates of predictions, which preceded the expected language units in the processing of single words (Pulvermüller et al., 2006; Dikker and Pylkkänen, 2013; Söderström et al., 2016), noun phrases (Fruchter et al., 2015), and sentences (Maess et al., 2016), although brain indexes of fine-grained semantic aspects of an expected utterance are still missing. A degree of semantic specificity is suggested by an anticipatory frontocentral potential, which emerges when subjects expect visual (Kilner et al., 2004) and acoustic (Grisoni et al., 2016) stimuli signifying body actions. This anticipatory activity resembles the readiness potential (RP), a brain indicator of intentions to move (Kornhuber and Deecke, 1965). Using high-density electroencephalography (128-channel EEG), we here report a semantic RP (SRP), which emerges during sentence processing and reflects aspects of the meaning of predictable action words before they appear.

To obtain contexts inducing specific action-semantic expectations, we created affirmative high-constraint (AHC) sentence fragments, upon which specific face- or hand-related action words were reported to follow with high probability. Control conditions displayed the same sentences with negation, thereby cancelling the expectation of specific words [negative low-constraint (NLC) sentences]. To separate brain indexes of predictability from those of negation (Tettamanti et al., 2008), we also investigated negative high-constraint (NHC) sentences, including predictable action words (Table 1).

**Table 1.** Experimental conditions and example stimuli

Conditions	Sentence fragments	SRP	Expected/Unexpected critical words	N400
AHC	I take the pen and I	**+**	Write/eat	−**/+**
	I find the broom and I	**+**	Sweep/smoke	−**/+**
	I take some grapes and I	**+**	Eat/write	−**/+**
	I find a cigarette on the desk and I	**+**	Smoke/sweep	−**/+**
NHC	I take the pen but I do not	**+**	Write/eat	−**/**−
	I find the broom but I do not	**+**	Sweep/smoke	−**/**−
	I take some grapes but I do not	**+**	Eat/write	−**/**−
	I find a cigarette on the desk but I do not	**+**	Smoke/sweep	−**/**−
NLC	I do not take the pen and I	−	Write/eat	**+/+**
	I do not find the broom and I	−	Sweep/smoke	**+/+**
	I do not take some grapes and I	−	Eat/write	**+/+**
	I do not find a cigarette on the desk and I	−	Smoke/sweep	**+/+**

Each of the three context conditions, AHC, NHC, and NLC, contained sentence fragments specifying hand or leg actions. The second column contains examples of the sentence fragments, which elicit different expectations of subsequent critical words. The next column shows whether the context of the sentence fragments licensed strong predictions on specific critical words, in which case an SRP was expected (+). If not, no SRP was predicted (−). The sentences were completed with either expected or unexpected critical words, which were either face or hand related and thus either body part congruent with the fragments or not. The last column shows whether an enlarged N400 was expected (+) or not (−), depending on the critical word presented.

HC contexts inducing expectations of specific words may specifically preactivate the neuronal circuits processing these words. Therefore, an SRP was expected in HC contexts but not in LC sentences. Crucially, if semantic aspects of predictable words are reflected before critical word onset, the SRP should differ between contexts predicting words with different meanings. We took advantage of previous works in which action words (e.g., “bite” vs “grasp”) activated their related body part representation in sensorimotor cortex (i.e., “mouth” vs “hand”; Hauk et al., 2004). Therefore, the SRP in AHC contexts may reflect specific semantic predictions by differentially activating sensorimotor cortex. In case negation is reflected in specific brain processes (Fischler et al., 1983; Nieuwland and Kuperberg, 2008), its effects may include the anticipation of multiple semantic alternatives (NHC condition); note that negation implies that the predictable proposition is not true, therefore giving rise to considering alternative action possibilities (Kühn and Brass, 2010), which may be manifest in broader sensorimotor activation.

The predictive semantic processes have further implications for the brain responses following the critical word (Holcomb and Neville, 1990). As no specific semantic prediction is possible in NLC sentences, all target words were semantically unexpected so that generally large N400 values were hypothesized. In contrast, the AHC condition led to specific action-semantic expectations, which were violated by incongruent endings, so that only these should lead to large N400 values. Because negated predictive contexts imply the processing of both target words and semantic alternatives, a further prediction was the reduction of the N400 to critical words in NHC contexts independent of sentence congruency (Table 1).

## Materials and Methods

### 

#### 

##### Participants.

Twenty-five healthy adults (mean age, 24.1 years; age range, 20–29 years; 14 females) participated after giving informed written consent. All participants were monolingual English native speakers who had not learned any second language before the age of 8 years. All participants had normal hearing, normal motor control, normal or corrected-to-normal vision, and no record of neurological or psychiatric disease. One participant was excluded due to excessively noisy EEG signals. Therefore, data from 24 participants (mean age, 24.1 years; age range, 20–29 years; 14 females), all of them strongly right handed as determined by the Oldfield handedness inventory (Oldfield, 1971; mean ± SD laterality quotient, 80.6 ± 14.9), were used for the EEG analysis. Participants provided written informed consent before participating in the study; procedures were approved by the Ethics Committee of the Charité Universitätsmedizin, Campus Benjamin Franklin, Berlin, Germany.

##### Stimuli and experimental design.

One hundred thirty-eight “congruent” English sentences were constructed and grouped into six categories of 23 sentences each on the basis of both the target verb they contained (i.e., face- or hand-related) and the sentence type and context in which the word was embedded (i.e., AHC, NHC, and NLC).

**Figure 1.**
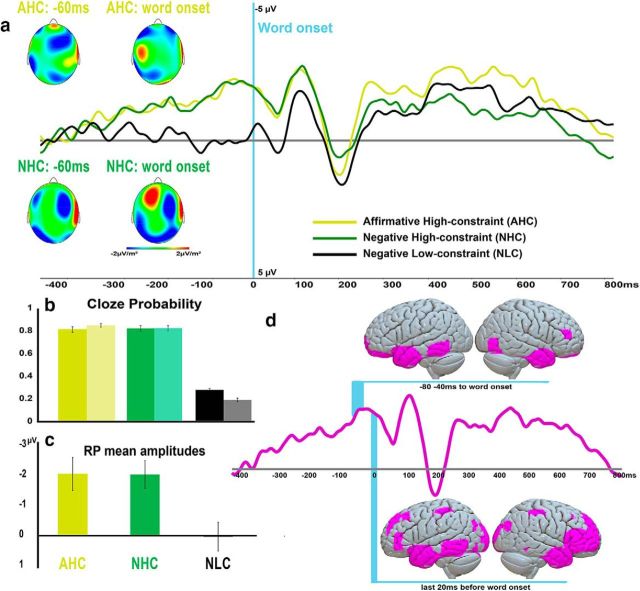
Cloze probability and electrophysiological results. ***a***, Event-related potentials elicited in the three context (AHC, yellow; NHC, green; and NLC, black) as the average of the frontocentral electrodes (FC1, FC2 FCz, FC3, FC4, C1, C2, Cz, C3, C4, CP1, CP2, CPz, CP3, and CP4). ***b***, Cloze probability evaluation of the experimental sentences. To estimate the predictability of our sentences, we followed established cloze probability tests taking the frequency with which the EEG and an independent group of participants reported, at least once, the word presented in the semantically congruent condition. From left to right, AHC (yellow: opaque for EEG participants, transparent for an independent sample), NHC (green: opaque for EEG participants, transparent for an independent sample), and NLC (black: opaque for EEG participants, transparent for an independent sample) contexts (mean and SEM). In ***c***, the RP mean amplitude (in microvolts) extracted from the last 100 ms before word onset are plotted. ***d*** shows the RP collapsed across the HC conditions (violet) together with the corresponding sources estimated at two different latencies (light blue highlighted; i.e., from 80 to 40 ms before word onset, and in the last 20 ms before word onset). All indicated clusters were significantly active (*t* tests, *p* < 0.05, whole-brain FWE correction).

First, triplets of semantically similar sentences were created whose final words were either face- or hand-related action words. Each triplet included one of the following sentence types: AHC, “I VERB PHRASE and I VERB…” (e.g., “I take the pen and I write”); NHC, “I VERB PHRASE but I do not VERB” (e.g., “I take the pen but I do not write”); and NLC, “I do not VERB PHRASE and I VERB” (e.g., “I do not take the pen and I write”; Table 1). The target words were selected on the basis of an evaluation performed by 10 English native speaker participants (mean ± SD age, 28.3 ± 5.19 years; 6 female), who did not take part in the EEG experiment. They were asked to complete fragments of each sentence missing the final verb in the sentence and list words they would expect in these contexts (cloze test). Participants had to read sentence fragments one by one and write up to three possible completions. The fragment order was randomized across participants. Data showed similar and hypothesis-based modulation of expected semantic types with the repeated-measures ANOVA revealing a main effect of the three-level factor context (*F*_(2,44)_ = 671.203, *p* < 0.001, ηp^2^ = 0.97; Fig. 1*b*). Bonferroni-corrected planned comparison tests revealed that the NLC sentences were completed with more uncertainty compared with AHC (*p* < 0.001) and NHC (*p* < 0.001) sentences, with no significant difference between the latter two (*p* = 1).

Target words were semantically related to the sentence fragments (e.g., pen–write), they had a predominant use as verbs and included one or two syllables; the two semantic word categories, face- and hand-related action words, were matched for mean word length (average ± SD of letters: face-related, 4.1 ± 0.81; hand-related, 4.6 ± 0.89) and standardized word frequency, computed as the logarithm of the number of occurrences of a word form within the British National Corpus (http://corpus.byu.edu/bnc/; face-related, 3.37 ± 0.51; hand-related, 3.44 ± 0.67; *t* = 0.37, *p* = 0.72).

Furthermore, the following features were implemented to exclude possible confounds: sentence contexts further constrained target words to be understood as verbs, as words of different grammatical category may elicit different ERPs (Nobre and McCarthy, 1995). All sentences were in first person singular present active form, as conjugation of action words may modulate cortical activity (Papeo et al., 2011). Sentences could be used as statements or reports; untypical words and nonliteral usage were avoided (i.e., technical terms, long compounds, proverbs, and idioms). Sentence length was matched between face- and hand-related sentences (average ± SD of words within the AHC context: face-related, 8.8 ± 1.7; hand related, 8.1 ± 1.3). Because it is difficult to find sufficient numbers of words with specific semantic features that are also matched for a range of psycholinguistic properties, it was necessary to repeat three words of each semantic type (i.e., face- and hand-related action words). Although word repetition may reduce the word-elicited brain response following the items (Sekiguchi et al., 2001), any such repetition-related ERP attenuation would affect both semantic word types to the same degree. Furthermore, no data are presently available that address repetition effects on the semantically predictive brain response appearing before critical word onset, which we first report here. If present, any repetition-related attenuation of anticipatory brain activity would work against finding neurophysiological correlates of semantic differences.

In addition to the sentences ending on target words (“congruent sentences”), we created 138 semantically incongruent sentences by exchanging the face- and hand-related critical words between contexts. Specifically, each face-related word was replaced in its context with a hand-related word similar in length, and vice versa. Therefore, the entire stimulus set consisted of 276 sentences.

We recorded multiple repetitions of all sentences uttered by a female native speaker of English and selected items that were acoustically similar (criteria: length, loudness, and intonation contour). The recordings of the critical words from the two semantic categories were matched for fundamental frequency (F0) and sound energy. Finally, the three types of sentence fragments were normalized to the same average sound energy calculated as root mean square power. The word recognition point (WRP; Marslen-Wilson, 1987) of the target words was estimated by a single native speaker of English, who was presented with gates of all critical words increasing in length in steps of 50 ms (Grosjean, 1980; Pulvermüller et al., 2003). The estimated WRP of a given word was assumed to lie at the gate length of the first correct and confident recognition (see Marslen-Wilson, 1987). The average word recognition point was computed as the average of all the face- and hand-related words. The WRP lay ∼450 ms after word onset and did not differ between semantic types (face-related words: average, 443 ms; SD, 142; hand-related words: average, 466 ms; SD, 93).

The EEG experiment consisted of one experimental block in which the 276 sentences were presented in random order to the participants. We created three separate lists, in each of which the sentences order was randomized; each EEG participant was randomly assigned to one of these lists. The interstimulus interval between the end of the sentence fragment constituting the context and the final (target or incongruent) word onset was 1500 ms. This pause was necessary to separate the neurophysiological response following the end of the sentence fragment from any predictive RP-like activity preceding the subsequent action verb. Note, however, that this pause did not lead to unnatural sounding sentences; hesitation phenomena and pauses naturally occur in spontaneous conversation. The intertrial interval between sentences was 3100 ms. The entire EEG recording lasted ∼25 min.

##### Apparatus and procedure.

The experiment was conducted in the electrically and acoustically shielded chamber of the Brain Language Laboratory, Freie Universität Berlin. Outside the chamber, one personal computer (PC)-controlled stimulus presentation, timing and randomization using E-Prime 2.0.8.90 software (Psychology Software Tools; RRID: SCR_009567). Inside the chamber, a separate PC was used to show a silent movie free of human action (“The Blue Planet,” BBC/Discovery Channel coproduction) to the participants, who were seated 1 m from the monitor. During EEG recording, all of the acoustic stimuli were presented binaurally through high-quality headphones (Ultrasone PRO 450, S-LOGIC). Participants were instructed to focus their attention on the movie, and they were told that the acoustic stimuli appearing during the film were of no relevance and should be ignored. After the EEG recording, all of the participants, seated in front of a PC, were asked to evaluate the entire stimulus set with a cloze probability test managed with E-Prime 2.0.8.90 software. Participants were instructed as follows: “You will hear several incomplete sentences. Please write down which words you would use to complete each sentence you hear. You can write one, two, or three possible completions with one or two words. If you do not have any idea, please don't write anything down.” Therefore, the participants had to listen to sentence fragments (i.e., the stimuli sentences without the target word) and write down the words they would expect in the respective contexts. Upon responding, they were presented with the next incomplete sentence. The sentence order was randomized among participants. After completion of this sentence evaluation, subjects were presented one by one with the target words from the study and the following semantic ratings had to be made: (1) “How strongly are the following words related to face actions?”; and (2) “How strongly are the following words related to hand/arm actions?” Participants had to listen to the stimuli and click, with the left button of the mouse, on a continuous visual analog scale (VAS) ranging from 0 (weak) to 100 (strong). The order of the words was randomized, as it was the order with which the two semantic ratings were administered to participants.

##### Electrophysiological recordings and preprocessing.

The EEG was recorded through 128 active electrodes embedded in a fabric cap (actiCAP 128Ch Standard-2, Brain Products) and arranged according to the international 10–5 system. Three electrodes (placed above and below the left eye and to the right outer canthus of the right eye) were used to measure vertical and horizontal electro-oculograms. All electrodes were referenced to an electrode placed on the tip of the nose. Data were amplified and recorded using the BrainVision Recorder (version 1.20.0003; Brain Products; RRID: SCR_009443), with a passband of 0.1–250 Hz, sampled at 1000 Hz, and stored on a disk. Impedances of all active electrodes were kept <10 KΩ. Off-line analysis started with data down-sampling to 250 Hz. Afterward, independent component analysis (ICA) with standard parameters for artifact removal, as implemented in EEGLAB 10 [Swartz Center for Computational Neuroscience (http://www.sccn.ucsd.edu/eeglab); RRID: SCR_007292] has been carried out. A component was considered to be artifactual when its topography showed peak activity only over the horizontal or vertical eye electrodes and when it showed a smoothly decreasing power spectrum (which is typical for eye movement artifacts (Delorme and Makeig, 2004). After calculating the independent components, eye blink components were removed from the EEG data. After ICA, off-line analysis was performed with Brain Products' Analyzer 2.0 (Brain Products; RRID: SCR_002356). The electrophysiological signal was filtered using a digital 20 Hz low-pass filter that is typical for slow brain potentials (Luck and Kappenman, 2012). Since the only two previous publications on perceptually induced RPs reported anticipatory activity starting ∼400 ms (Kilner et al., 2004) and 250 ms (Grisoni et al., 2016) before the expected perception, trials were epoched from 480 ms before word onset to 840 ms after. The first 50 ms of the segmentation were used as baseline. Note that studies with overt motor responses sometimes show much earlier RP onsets (Kornhuber and Deecke, 1965), so that action-related perceptions (of pictures or sounds) produced comparatively short RPs. Consistently, preliminary analysis of our present data indicated the first RP-like deflection at ∼400 ms before critical word onset. Epochs with voltage fluctuation of >100 μV and those contaminated with artifacts due to amplifier clipping, bursts of electromyographic activity, or alpha power were excluded from averaging by a semiautomatic rejection procedure. On average, ∼10% of the trials were rejected because they violated these artifact criteria.

#### Data analysis

##### Stimulus ratings.

The predictabilities of critical words to appear after the sentence fragments was defined as the proportion of participants who correctly named the critical word when being presented with the fragment. A 2 × 3 repeated-measures ANOVA with the factors word type (face and hand related) and context (AHC, NHC, and NLC) was performed on these frequencies. The visual analog scale scores for the two target word categories (i.e., face- and hand-related words) were analyzed by means of a 2 × 2 repeated-measures ANOVA with the factors VAS (face and hand relatedness) and word type (face- and hand-related words).

##### Prestimulus anticipatory activity.

For investigating predictive brain activity in anticipation of action words, the time window of interest ends at the onset of these critical stimuli. The RP develops gradually within several hundreds of milliseconds, with premotor and primary motor activation appearing in its very last part, within ≤100 ms before movement or critical stimulus onset (Erdler et al., 2000; Kilner et al., 2004; Edwards et al., 2010; Grisoni et al., 2016). In a sound perception paradigm (Grisoni et al., 2016), we recently found most pronounced somatotopic RP effects during the last tens of milliseconds before predicted sound onset. Therefore, we extracted the mean ERP amplitudes (in microvolts) for the last 100 ms and for the last 20 ms immediately before critical word onset at central electrodes (FC1, FCz, FC2, C1, Cz, C2, CP1, CPz, and CP2), where the RP is known to be largest (Deecke et al., 1969). First, we tested whether the negative deflection observed was significant in the three contexts. To this end, the average of the mean ERP amplitudes (in microvolts) obtained for the two word type expectations (i.e., face- and hand-related words) in each of the three contexts (i.e., AHC, NHC, and NLC) were submitted to separate *t* tests against zero. Subsequently, we performed a 3 × 2 repeated-measures ANOVA with the factors context (AHC, NHC, and NLC) and expected semantic type (face- and hand-related words). We also investigated possible neurophysiological changes across the experiment by directly comparing the first 12 trials with the last 12 trials in each context, by calculating a 2 × 3 repeated-measures ANOVA with the factors trials (first, last) and context (AHC, NHC, and NLC). Note that this comparison is important for addressing the possibility of an influence of experiment-specific processing strategies (Neely, 1977), which may develop during the study. In case significant interactions were found, topographical differences between face- and hand-related word contexts were investigated using a larger array of frontoparietal electrodes (F7, F3, Fz F4, F8; T7, C3, Cz, C4, T8; P7, P3, Pz, P4, and P8). Because the NLC condition did not show reliable RPs, this evaluation focused on the predictable contexts (i.e., AHC and NHC, which both produced clear RPs; Luck and Kappenman, 2012). In this case, a 2 × 2 × 3 × 5 repeated-measures ANOVA design included the factors gradient (anterior–posterior, three levels), laterality (left–right, five levels) along with context (AHC and NHC) and expected semantic type (face- and hand-related words).

##### fMRI and source localization.

Because our main predictions addressed cortical areas relevant for semantic prediction, it was crucial to estimate and compare the sources of the observed neurophysiological responses. Therefore, ERP topographies showing significantly different activation patterns between contexts and expected semantic types were further analyzed by calculating distributed cortical sources using standard methods implemented in SPM8 (Litvak et al., 2011), which had previously been used in our laboratory (Grisoni et al., 2016). As any distributed source localization, this method cannot overcome the nonuniqueness of the inverse problem (von Helmholtz, 1853) but successfully uses established priors for providing plausible source solutions for cognitive experiments. The template structural MRI included in SPM8 was used to create a cortical mesh of 8196 vertices, which was then coregistered with electrode cap space using the following three electrodes as fiducials: Fpz, TP9, and TP10. The volume conductors were constructed with an EEG (three-shell) boundary element model. The averaged RP responses were then inverted at the group level, using the multiple sparse prior technique, specifically the “Greedy Search” algorithm (Litvak and Friston, 2008). To achieve good signal-to-noise ratios (SNRs) in estimating cortical sources in each participant for each expected semantic type, source images from the two contexts (i.e., AHC and NHC) were averaged. The same procedure was used for localizing context effects, thus collapsing face- and hand-related activation maps across the AHC condition and, again, for the NHC context. Activation maps were then smoothed using a Gaussian kernel of full-width at half-maximum (FWHM) of 14 mm, resulting in four images per participant (i.e., face- and hand-related expected words; AHC and NHC contexts). To test whether the face- and hand-related expected semantic types differed between each other within the sensorimotor cortices, we performed voxelwise paired *t* tests in predefined regions of interest (ROIs).

As some of the predictions addressed activity in the motor system, two motor ROIs were defined based on the results of a separate fMRI localizer experiment, which was performed with different subjects. To this end, a group of 31 participants (mean age, 23.2 ± 5.3 years; 16 females; mean laterality quotient, 91.7 ± 15.3; selected with the same criteria as for the EEG experiment) performed lip and hand movements (Hauk et al., 2004). Participants were scanned in a 3 T Siemens Tim Trio Scanner system. The brain regions were defined in relation to a baseline in which the participants were resting. Participants had to perform repetitive lip movement, avoiding contact between the lips, and finger movement with the right index finger, avoiding contact between finger and hand. Each movement block was 15 s long and repeated four times, with 15 s of rest between blocks. Block order was random. The “peak activation voxel” (largest *t* value) in frontocentral cortex was selected per movement. The lips movement > baseline contrast revealed activity located in a ventral pre-central region [MNI coordinates: −54, −10, 39; *p* < 0.001, familywise error (FWE) corrected at the whole-brain level], whereas the hand movement > baseline contrast revealed activity located in a dorsolateral pre-central region (MNI coordinates: −36, −18, 62; *p* < 0.001, FWE corrected).

ROIs for the ERP generator localization were created with Marsbar 0.43 (MARSeille Boîte À Région d'Intérêt SPM toolbox; RRID: SCR_009605) and defined as 14 mm-radius spheres (i.e., matching the FWHM of the smoothing parameter) centered at the above-mentioned coordinates. These ROIs were then combined in a unique mask image that was used as an explicit mask. Only voxels included in this mask were considered when comparing the sources of face- and hand-related expected semantic type conditions. Similarly, differences between AHC and NHC contexts within and outside the sensorimotor cortices were tested by means of two sets of paired *t* test. A first comparison was performed on the whole brain, whereas a second hypothesis-driven analysis was tested for specific differences within the sensorimotor system. To this end, we created a mask image that included Brodmann areas (BAs) 1–4 and 6 (i.e., primary motor, premotor, and somatosensory areas, respectively) using the WFU_PickAtlas (Maldjian et al., 2003). Finally, to test the temporal development of the RP, we performed source estimations, using the same methodology, on the ERP obtained by averaging all of the high-constraint conditions together. We extracted the ERP activation maps from two nonoverlapping time windows, the interval around the maximum of the 100 ms-window before critical word onset (from 80 to 40 ms) and the terminal 20 ms window. Thus, for each participant we obtained two images (i.e., one for each time window) that were submitted to *t* tests against zero. For fMRI and all the source analyses (*t* tests), *p* values were thresholded at *p* < 0.05 corrected for multiple comparisons using the FWE procedure; significant clusters were considered only if they included >60 voxels.

##### Poststimulus potentials.

Two word-related potential components were analyzed, the early negative-going peak at ∼100 ms (N100) and the subsequent N400. Since electrophysiological poststimulus responses are usually reported with a baseline correction computed across the last 100 ms before word onset (Penolazzi et al., 2007), we adopted the same procedure.

##### N100.

First, we assessed the N100 responses on frontocentral electrodes (F1, Fz, F2, FC1, FCz, and FC2), where this early component is known to be largest (Vaughan and Ritter, 1970) and therefore the best SNR can be expected. The N100 response was calculated as the mean amplitude in the 40 ms time window centered at 106 ms from word onset. This latency was obtained as the local maximum (within the interval 0–200 ms from word onset) of the grand average obtained by collapsing all of the conditions together. Potential effects of word and context were assessed with a 3 × 2 × 2 repeated-measures ANOVA with the factors context (AHC, NHC, and NLC), critical word type (face- and hand-related words), and congruency (congruent and incongruent with respect to context-induced expectations).

##### N400.

Since critical words were presented acoustically and the word recognition point of these items was estimated to be ∼450 ms after their onset, we expected a late N400-like response (500–600 ms from word onset). The mean amplitudes extracted from three anterior–posterior midline electrodes (FCz, CPz, and POz), canonical sites for the N400 (Penolazzi et al., 2007), were submitted to a 3 × 2 × 2 × 3 repeated-measures ANOVA with the factors context (AHC, NHC, and NLC), semantic word type (face- and hand-related words), congruency (congruent and incongruent with respect to the expectations), and gradient (anterior–posterior; electrodes FCz, CPz, and POz). Additional analyses performed on broader frontoparietal electrode selections confirmed the results obtained.

For further investigation of any significant interaction effects revealed by the ANOVAs, *F* tests were used for planned comparisons. All results reported survived a Bonferroni correction. Partial η-square values (ηp^2^) are reported as indexes of effect sizes. When sphericity violations were found in the ANOVAs, a Greenhouse–Geisser correction was applied, and corrected *p* values are reported.

## Results

### Stimulus ratings

Participant ratings confirmed that affirmative and negative high-constraint sentence contexts were comparable with regard to the probabilities with which their critical congruent words could be determined from sentence context; in contrast, the low-constraint conditions were confirmed to include unexpected critical words. The repeated-measures ANOVA revealed a main effect of the three-level factor context (*F*_(2,44)_ = 712.58, *p* < 0.001, ηp^2^ = 0.97). Bonferroni-corrected planned comparison tests revealed that the NLC sentences were completed with more uncertainty compared with AHC (*p* < 0.001) and NHC (*p* < 0.001) sentences, with no significant difference between the latter two (*p* = 1; Fig. 1*b*). The VAS scores assessing the semantic face and hand relatedness of our words revealed main effects of the VAS (*F*_(1,23)_ = 12.68, *p* = 0.002, ηp^2^ = 0.35), due to higher scores in the face-relatedness compared with the hand-relatedness VAS ratings (*p* = 0.002), and word type (*F*_(1,23)_ = 34.57, *p* < 0.001, ηp^2^ = 0.60), due to higher scores for the face-related compared with hand-related words. Crucially, the crossover interaction of the VAS and word type (*F*_(1,23)_ = 295.95, *p* < 0.001, ηp^2^ = 0.93) was significant due to higher scores for the face-related words compared with the hand-related words in the VAS assessing face relatedness (*p* < 0.001) and, vice versa, higher scores for hand-related words compared with face-related words in the VAS assessing hand relatedness (*p* < 0.001).

### Sentence contexts predicting action-related words and concepts elicit anticipatory activity reflecting semantic expectation before critical word onset

**Figure 2.**
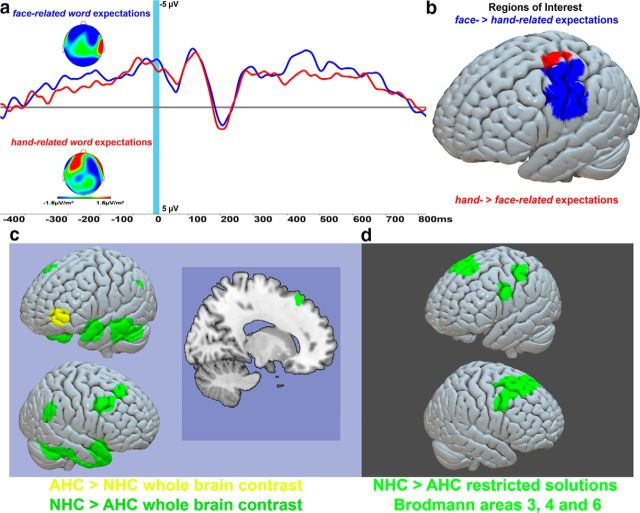
SRP: predictive brain activity for face-related and hand-related words. ***a***, SRP curves in anticipation of face-related (blue) and hand-related (red) words (high-constraint contexts collapsed). The light blue window shows the last 20 ms before word onset. ***b***, Source analysis results comparing predictive brain activity for face-related and hand-related words within the sensorimotor cortex (see Materials and Methods). Ventral regions (blue) revealed a significant contrast face-related > hand-related word predictions, whereas dorsolateral sources showed the opposite contrast (red). ***c*** shows statistically significant clusters obtained after whole-brain FWE correction AHC > NHC (yellow) and NHC > AHC (green). The latter contrast (green) showed activity in TOM areas and (i.e., temporal lobes, temporoparietal junction, and frontal and medial frontal cortex) as well as (see ***d***) widespread sensorimotor system activity.

Both HC conditions elicited a slowly emerging negative-going potential starting ∼350 ms before appearance of the critical word stimulus (Figs. 1*a*,*c*, 2*a*). The smoothly growing shape of the waveforms and their frontocentral scalp distribution (Kornhuber and Deecke, 1965) are consistent with the RP profile. In the last 100 ms before critical word onset, only the high-constraint conditions elicited reliable RPs, as documented by *t* tests against zero (AHC: mean amplitude = −2.05 μV, *t*_(23)_ = −3.72, *p* = 0.001; NHC: mean amplitude = −2.04 μV, *t*_(23)_ = −4.42, *p* = 0.0002; NLC: mean amplitude = 0.02 μV, *t*_(23)_ = 0.05, *p* = 0.96, n.s.). Repeated-measures ANOVA performed on this time window revealed a significant main effect of context (*F*_(2,46)_ = 6.61, ε = 0.95, adjusted *p* = 0.003, ηp^2^ = 0.22) with Bonferroni-corrected planned comparison tests revealing that the NLC context induced weaker anticipatory activity compared with AHC (*p* = 0.008) and NHC (*p* = 0.009) contexts, with no significant difference between the latter two (*p* = 1). The repeated-measures ANOVA comparing the RP amplitudes in the three contexts extracted for the first and the last 12 trials of the experiment confirmed the significant main effect of context (*F*_(2,46)_ = 4.33, ε = 0.98, adjusted *p* = 0.019, ηp^2^ = 0.16) observed on the whole dataset but gave no evidence of a change of RP signatures across the experiment; this result fails to confirm a physiological manifestation of experiment-induced strategies developing across the study (see Materials and Methods). Repeated-measures ANOVA on a larger array of frontoparietal electrodes at the end of the RP curves and before word onset revealed a main effect of laterality (*F*_(4,92)_ = 3.18, ε = 0.59, adjusted *p* = 0.042, ηp^2^ = 0.12) with Bonferroni-corrected planned comparison revealing a topographical distribution consistent with the RP profile (Shibasaki and Hallett, 2006) in right-handed participants, where the central electrodes show larger amplitudes compared with most right-hemispheric recording sites (*p* = 0.02) but not relative to the most left-lateral ones (*p* = 1). Furthermore, the anticipatory activity was modulated in its topographical distribution by the semantic type of the expected words (i.e., face or hand related) as revealed by the significance of the interactions among the factors expected semantic type, gradient, and laterality (*F*_(8,184)_ = 3.7, ε = 0.37, adjusted *p* = 0.015, ηp^2^ = 0.14). Finally, the context also affected the ERP topographies as revealed by a context × gradient (*F*_(2,46)_ = 4.57, ε = 0.73, adjusted *p* = 0.027, ηp^2^ = 0.17) interaction. These results demonstrate that semantic features of the context are manifest in the anticipatory potential, which we therefore call the SRP.

Distributed sources underlying the SRP were calculated to determine its cortical generators. First, we investigated source estimations for SRP collapsed across all predictable conditions. *t* tests against zero revealed generators located in areas traditionally associated with semantic processing, including the anterior temporal areas and the inferior prefrontal cortex (Bookheimer, 2002; Patterson et al., 2007; BAs 45/46; Fig. 1*d*). Comparisons between contexts revealed generator clusters specific to the expected semantic type, which were located in the somatosensory and motor areas bilaterally and in dorsolateral prefrontal cortex (BA 9; Table 2, Fig. 1*d*). To better disentangle this complex pattern of activations, SRP sources were compared between affirmative and negated contexts (i.e., AHC vs NHC) and between specific semantic expectations (i.e., face- vs hand-related actions). Just before word appearance, where ERP data had indicated topographical dissociations between expected semantic type and contexts, the whole-brain AHC > NHC contrast revealed a significant cluster located in the left inferior frontal region, whereas the opposite NHC > AHC contrast revealed significant clusters located in temporal pole, temporoparietal junction (TPJ) and dorsolateral prefrontal cortex (BAs 8 and 9; Fig. 2*c*, Table 2). The same contrast restricted to sensorimotor areas (i.e., BAs 1–4 and 6; see Materials and Methods) revealed a more widespread motor activation in the NHC compared with the AHC context (Fig. 2*d*, Table 2). The reverse contrast did not yield significance.

**Table 2.** fMRI and source analysis results

	MNI coordinates			*t* values (peak level)	Number of voxels	*p* values (FWE corrected)	Brodmann areas	Cortical areas
	*x*	*y*	*z*					
fMRI: motor localizer results: Lips movement > baseline	−54	−10	39	12.39		<0.001	4	Ventral Pre- and Post-central gyrus
fMRI: motor localizer: Fingers movement > baseline	−36	−18	62	16.66		<0.001	4	Dorsal Pre- and Post-central gyrus
*t* test against zero on sources from the ERP obtained by collapsing all the high-constraint conditions first time window: −80 −40 ms before word onset	52	−6	−30	8.87	4039	0.015	20	Anterior temporal lobe
	−44	16	−26	7.62	3827	0.016	38	Anterior temporal lobe
	−18	50	−16	6.25	3288	0.017	11	Orbitofrontal cortex
	4	56	−14	6.13	580	0.036	11	Orbitofrontal cortex
	−46	−50	−14	4.52	1462	0.027	20	Posterior temporal lobe
	44	44	14	4.15	80	0.046	45	Posterior inferior prefrontal cortex
*t* test against zero on sources from the ERP obtained by collapsing all the high-constraint conditions second time window: last 20 ms before word onset	−48	−12	−30	8.61	6093	0.007	20	Anterior temporal lobe
	52	−10	−32	8.14	3187	0.014	20	Anterior temporal lobe
	42	36	−2	6.45	7343	0.005	47	Posterior inferior prefrontal gyrus
	16	−94	14	5.19	2677	0.016	18	Occipital cortex
	−30	24	40	5.06	2013	0.019	9	Dorsolateral prefrontal cortex
	46	−28	46	5.00	1100	0.026	3/4	Pre- and post-central gyrus
	−44	−30	48	4.06	1137	0.026	3	Dorsolateral Pre- and Post-central gyrus
	−42	40	2	4.71	476	0.035	45	Inferior prefrontal cortex
	38	−78	28	4.32	161	0.042	39	Parietal lobe
	56	−46	−10	4.21	67	0.045	20	Posterior temporal lobe
	−40	−76	28	4.16	167	0.042	39	Parietal lobe
Face- > hand-related expected semantic types ROIs	−50	−22	44	4.71	1212	0.021	4	Pre-central gyrus
	−42	−22	50	4.19	508	0.031	3	Post-central gyrus
Face- > hand-related expected semantic types ROIs	−24	−24	66	4.77	99	0.042	4	Pre-central gyrus
Whole-brain contrast: AHC > NHC	−40	58	4	4.17	157	0.027	46	Inferior frontal gyrus
Whole brain contrast: NHC > AHC	44	−22	−26	9.29	6935	<0.001	20	Temporal pole
	−52	−22	−26	9.29	4650	<0.001	20	Temporal pole
	52	−42	36	6.00	391	0.016	48	Temporoparietal junction (TPJ)
	32	16	38	5.70	657	0.010	46	Dorsolateral prefrontal cortex
	−50	−46	34	5.53	152	0.027	48	Temporoparietal junction (TPJ)
	16	30	52	5.43	124	0.029	8	Medial frontal cortex
Brodmann areas 3, 4, and 6 restricted contrast: NHC > AHC	12	30	58	5.74	1431	0.014	6	Pre-central gyrus
	34	4	34	5.38	117	0.039	6	Pre-central gyrus
	−8	28	44	4.84	339	0.031	6	Pre-central gyrus
	−50	4	36	4.47	125	0.039	6	Pre-central gyrus
	−54	−24	56	4.25	180	0.036	3	Post-central gyrus

For all significant contrasts calculated on the fMRI results and the cortical sources of the first and second SRP time intervals and for all significant clusters, the table displays the MNI coordinates of the voxel with highest *t* value, its *t* value, the number of significant voxels per each significant cluster, FWE-corrected *p* value, and the Brodmann area labels where the “peak voxel” was found, along with a description of the cortical area where the active cluster was located.

When testing for semantic specificity of the RP brain generators, whole-brain corrected comparisons were not significant. Hypothesis-driven focus on ROIs in the motor system, namely on face and hand representations, showed relatively larger activation in the ventrolateral pre-central areas for contexts predicting face-related word and the reverse, greater activation for hand-related compared with face-related item expectation, in dorsolateral pre-central areas (Fig. 2*b*, Table 2).

### N400 values elicited by critical words show interactive effects of sentence polarity and prediction matching

**Figure 3.**
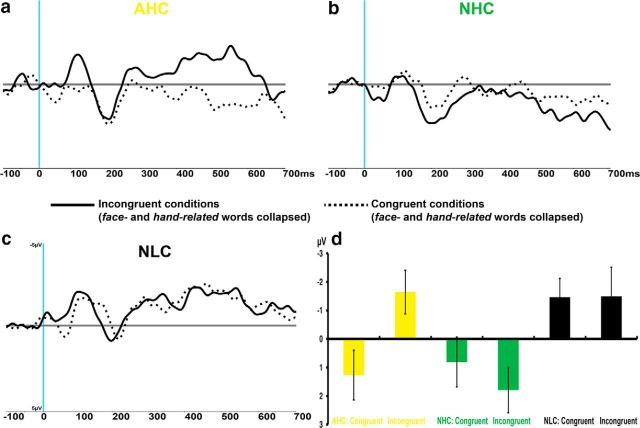
N400 results for the AHC, NHC, and NLC contexts. Congruent (dotted line) and incongruent (solid line) in AHC (top left, ***a***), NHC (top right, ***b***), and NLC (bottom left, ***c***) are plotted as the average of three midline electrodes used for statistical comparisons (FCz, CPz, and POz). Panel ***d*** (bottom right) shows the statistically significant interaction of the factors context and congruency [mean and SEM; from left to right affirmative high-constraint (yellow), negative high-constraint (green), and negative low-constraint (black) contexts].

Because much previous research has found neurophysiological effects of critical words violating context-induced semantic predictions in the N400 component (Kiefer and Martens, 2010; Fig. 3*a*,*d*), ERPs elicited by the critical words were calculated relative to a 100 ms baseline before critical word onset, as is the standard practice (Kutas and Federmeier, 2011). Figure 3 shows that the word-elicited potentials included an early positive deflection followed by a N100, a-positive going wave maximal at ∼200 ms, and a subsequent negative-going deflection (N400). Significant differences were absent for the early components, possibly due to acoustic variance across spoken word onsets. N400 mean amplitudes revealed a main effect of context (*F*_(2,46)_ = 4.73, ε = 0.88, adjusted *p* = 0.017, ηp^2^ = 0.17) with Bonferroni-corrected planned comparison tests showing that NLC contexts induced larger N400 responses than NHC contexts (*p* = 0.01) but were not relative to AHC conditions (*p* = 0.48), with no significant difference between the latter two (*p* = 0.32). Critically, there was a significant interaction between the factors context and congruency (*F*_(2,46)_ = 4.12, ε = 0.84, adjusted *p* = 0.029, ηp^2^ = 0.15), with planned comparison tests revealing that the expectancy violation in the AHC context produced a larger N400 response than expected congruent critical words (*p* = 0.03), whereas NHC or NLC contexts did not reveal any N400 modulation by word expectancy (both *p* = *1*; Fig. 3*d*). Crucially, whereas NLC contexts elicited large N400 values throughout, the N400 response was virtually absent after NHC sentence fragments. Specifically, the NHC incongruent condition elicited a significantly smaller negative-going response in the N400 interval compared with both AHC (*p* = 0.006) and NLC contexts (*p* = 0.01), with no significant difference between the latter two (*p* = 1). These results are evidence for true prediction violations in the incongruent AHC condition and both NLC conditions, but not in any of the NHC contexts. It appears that there is no truly unexpected word in the latter case, possibly because subjects were entertaining alternative hypotheses. These results confirm the prediction based on the hypothesis that multiple alternatives are activated in negated predictable sentence processing.

## Discussion

This study shows the emergence of a readiness potential (RP) in complex sentence contexts before semantically predictable words but not before unpredictable ones. Intriguingly, different semantic readiness potential (SRP) topographies and different cortical source constellations were found in anticipation of words with different meanings, therefore proving the presence of specific semantic information in predictive brain activity before the anticipated meaningful symbols appeared. Predicted words related to actions typically performed with different effectors (face and hand) elicited anticipatory brain activity whose sources were located in their corresponding sections of the sensorimotor cortex, thus revealing a pattern of semantic somatotopy (Pulvermüller et al., 2005; Kemmerer, 2015). Semantically constrained contexts led to SRPs regardless of whether they had a positive or negative meaning, thus predicting a specific lexical item with high probability. Intriguingly, predictive sensorimotor activation was more widespread in negated than in affirmative high-constraint sentences, which is consistent with predictive processing of multiple semantic alternatives during the former. Furthermore, compared with affirmative sentence contexts, negated ones led to predictive activation in the temporoparietal junction, temporal pole, and the medial prefrontal cortex.

The hypothesis that multiple semantic alternatives are processed in the prediction of negated propositions is also consistent with the pattern of N400 responses to the congruent and incongruent critical words. Indeed, the typical pattern of a relatively enhanced N400 to semantically incongruous endings (Kutas and Federmeier, 2011) was found only for incongruous target words in affirmative sentence contexts. The low-constraint contexts produced similar N400 values for the semantically related and unrelated target words, as they were both unpredictable. Importantly, minimal N400 values were found in all high-constraint negative contexts, even for unexpected target words, thus suggesting that untypical action-semantic sentence endings were at some level expected in this type of context.

We focused here on sentences in which the predicted symbols were action verbs with a semantic relationship to overt bodily actions typically performed by human subjects, and it may be that aspects of our findings are specific, or most pronounced, for this lexical type. Furthermore, to avoid the overlap of brain responses elicited by sentence context and expected semantic types, we introduced a 1.5 s pause before the critical word, which may be seen as making language use in this experiment somewhat “unnatural.” In this view, the pause could lead to experiment-specific strategic processes, thus suggesting prudence in generalizing our results to natural language use. However, at least three arguments speak against this possibility. First, pauses naturally and frequently occur in normal conversation (Fernald et al., 1989); second, participants were listening to sentences passively while watching a silent movie, being instructed to ignore any verbal input, which works against an explicit strategy of stimulus analysis; and third, the comparison between the SRP mean amplitude extracted from the first and the last trials failed to indicate neurophysiological changes, although in case of the dependence of neurophysiological responses on experiment-specific strategies one would expect development of such strategies/responses across the experiment. Still, we cannot exclude that specific features of our experiment play a role in eliciting the predictive brain responses observed, and future work is therefore necessary to confirm and extend the present results. However, major features of the observed SRP dynamics are explainable by predictability and negation alone. For example, when the negation was placed at the beginning of the sentences, as in “I do not take the pen and I write,” we observed a drop in the ability to predict the final word “write” (Fig. 1*b*) and no anticipatory activity before the word (Fig. 2*a*). However, when the negation was placed just before the critical word (as in “I take the pen but I do not write”), the participants were still able to predict the congruent critical words just as in the affirmative sentence conditions (i.e., “I take the pen and I write”; Fig. 1*b*). Coherently, in these predictable situations we observed similar SRPs whose latencies, scalp distributions, and negative polarities are consistent with an RP (Fig. 2*a*,*c*).

### SRP as an index of semantic prediction

A broad range of studies on semantic processing investigated the N400 component (Kutas and Federmeier, 2011) to draw conclusions about the preactivation of lexicosemantic circuits during sentence comprehension (Van Berkum et al., 2005). However, as the N400 follows the critical predicted/unpredicted word, it does not directly reflect the buildup of activation related to prediction, but rather the match or mismatch between such predictions and the (expected or unexpected) critical stimulus. For example, DeLong et al. (2005) demonstrated that the sentence “The day was breezy so the boy went outside to fly an airplane” elicited larger N400 responses to the final article “an” and the noun “airplane” compared with the same sentence presented with the expected ending composed by the article “a” and the noun “kite.” The larger N400 elicited by the last noun (i.e., airplane) could be explained both in terms of word preactivations and semantic integration process, because the two nouns (i.e., “airplane” and “kite”) differ in meaning. However, the two articles “a” and “an” are meaningfully equivalent, thus revealing that the N400 responses in this case would imply that the listeners have already formed the expectation for “kite” rather than for “airplane” (Otten et al., 2007). Although these results show a brain index of semantic anticipation in sentence understanding, they still report responses following items that had first been predicted at an earlier stage—as even the determiners “a” or “an” were predictable based on the preceding sentence fragment.

Few studies address the direct neurophysiological correlates of predictions preceding expected language units. For example, Pulvermüller et al. (2006) reported MEG activity indexing the point in time of whole-word recognition before the end of spoken words, and Söderström et al. (2016) reported that brain activity elicited by word-initial phonemes indicates the predictability of the unfolding words. Dikker and Pylkkänen (2013) found brain correlates of the predictability of nouns upon corresponding object picture presentation, although picture makeup and repetition represent possible confounds of this work. Fruchter et al. (2015) found brain activation in response to adjectives related to their predictive information on subsequent nouns and localized the origin of this predictive activity in anterior temporal cortex. Maess et al. (2016) found brain responses reflecting the predictability of nouns upon stimulus verbs. On the background of these innovative studies of linguistic-predictive brain activity, an important novel finding of the present work was that, before predicted words appeared, anticipatory patterns of brain activity and their underlying source constellations revealed aspects of the meaning of the expected words and sentence endings. This bolsters the semantic character of the SRP. The word-related topography modulation was observed at the end of the RP curves when precentral gyrus activation and somatotopic differences are normally present in RP studies of voluntary movement tasks (Ball et al., 1999; Weilke et al., 2001). Furthermore, at the same latency the neurophysiological source analysis confirmed the hypothesis of different cortical generators between the face- and hand-related semantic expectations. As in earlier work (Hauk et al., 2004; Pulvermüller and Fadiga, 2010), semantically related somatotopic activity was observed; that is, the expectation of hand-related words brought about greater activation in hand motor areas compared with expectations of face-related words and, vice versa, greater activation within face motor areas when participants expected face-related compared with hand-related words (Fig. 2*a*,*b*, Table 2).

### Negative predictable contexts lead to processing multiple semantic alternatives

Previous studies of negation differed in their results. Fischler et al. (1983) found that N400 responses depended on semantic relationships between context and critical words, but affirmative or negated sentence meaning was reflected only at a later stage. However, recent investigations (Nieuwland and Kuperberg, 2008) showed larger N400 values for false statements compared with true statements, independent of negation (along with a further influence of pragmatic factors). Our present SRP sources confirm an early onset of sentential negation processing, possibly even before the final word in the sentence. In our results, unpredictable negated sentences elicited substantial N400 values, which were similar to those obtained when semantic expectations were violated (incongruent AHC condition). Therefore, the predictability and negation factors are both necessary to explain N400 dynamics following the critical words.

The simultaneous processing of multiple alternative action hypotheses offers an explanation why, in negated high-constraint contexts, SRP sources were more widespread and N400 values were generally minimal. Negation-related SRPs were due to larger sources not only in motor systems (see below) but also in temporoparietal junction, temporal pole, and the medial prefrontal cortex. These regions are part of the theory-of-mind (TOM) network (Saxe et al., 2004; Amodio and Frith, 2006). Although temporal poles and the left inferior posterior temporal cortex are frequently discussed as semantic hubs (see, for example, Patterson et al., 2007), the dorsal medial prefrontal cortex is not a semantic area in this sense. Regarding parietal cortex, particularly the TPJ, opinions are mixed, with some authors (Binder and Desai, 2011) attributing a general semantic role and others (Patterson et al., 2007) denying it. Therefore, we believe that the set of areas found active in negation processing is best characterized as similar to the TOM network, although it includes established “semantic areas.” Our suggestion that multiple action-semantic alternatives are processed in predictable negated contexts is consistent with a greater engagement of TOM networks and semantic systems of the human brain, including motor areas.

Previous studies reported diminished motor activity following action-related words in negative contexts (Tettamanti et al., 2008; Tomasino et al., 2010; Liuzza et al., 2011; Aravena et al., 2012). Our present results offer a possible explanation for why this was so: because, in some of the experiments, the action hypotheses had already been present in the predictive baselines, the neuronal circuit of the subsequent action concept was already primed so that reduced semantic activity, manifest in part in motor systems, was observed in response to word presentation. In a previous study, we showed that motor cortex activation to action words is indeed greatly reduced in contexts where these words are predictable so that their neuron circuits have been primed semantically by context (Grisoni et al., 2016). The current results shed new light on the brain mechanisms of semantic processing as they draw attention to the importance of the interplay between predictive processes and prediction resolution, here directly reflected by SRP and N400 responses, respectively. Furthermore, they demonstrate the importance of the specific and complementary roles of modality-specific and modality-general brain systems in sentence-level meaning processing.
